# Eco-Friendly Adhesion of Isosorbide-Based Polycarbonate

**DOI:** 10.3390/molecules30132843

**Published:** 2025-07-03

**Authors:** Ruiqi Han, Kazuaki Matsumura, Masayuki Yamaguchi

**Affiliations:** Graduated School of Advanced Science and Technology, Japan Advanced Institute of Science and Technology, 1-1 Asahidai, Nomi 923-1292, Ishikawa, Japan; s2320427@jaist.ac.jp (R.H.); mkazuaki@jaist.ac.jp (K.M.)

**Keywords:** adhesion, isosorbide, polycarbonate, biodegradable, poly(vinyl alcohol)

## Abstract

We investigated the practical adhesion of a conventional poly(vinyl alcohol) glue with a glassy isosorbide-based polycarbonate (ISB-PC) comprising isosorbide and 1,4-cyclohexanedimethanol. The addition of 1 wt.% of a copolymer of vinyl alcohol and butenediol to the ISB-PC greatly improved its lap-shear strength. This improvement may be attributed to the dissolution of the copolymer chains in the ISB-PC, which had a low water droplet contact angle. Furthermore, the blend was transparent because most of the copolymer chains dissolved in the ISB-PC. Microplastics present a serious environmental issue, even for adhesives. Therefore, the present technique to modify ISB-PC to show good lap-shear strength with a biodegradable glue is attractive.

## 1. Introduction

Various adhesives have been developed for plastics. They can be roughly classified as solution- or emulsion-type resins, reactive resins, and hot-melt adhesives [1,2]. Owing to the recent increasing awareness regarding the detrimental effects of microplastics on the environment, it is necessary to replace current adhesives with those that are biodegradable in soil and marine environments [3,4]. Furthermore, the use of organic solvents, which may cause sick building syndrome, is increasingly disparaged [5]. Certain natural polymers, such as starch, dextrin, and gelatin, are attractive as water-soluble and biodegradable adhesives. However, their practical adhesion regarding most plastics is significantly low. Poly(vinyl alcohol), which is not a natural resource, also shows biodegradability even in marine environments [6,7,8,9] and used as a glue [1,2,3,4,9]. However, its adhesion regarding most plastics is similar to that of the natural adhesives mentioned above, i.e., very low.

In the present study, we demonstrated that a conventional poly(vinyl alcohol) glue shows good adhesion with a specific plastic, i.e., isosorbide-based polycarbonate (ISB-PC), containing a small amount of another polymer.

ISB-PC has been commercialized as a biomass-based transparent plastic. It is widely considered an excellent candidate for the replacement of bisphenol-A polycarbonate and polymethylmethacrylate because it is non-toxic and has excellent mechanical and optical properties [10,11,12,13,14]. Therefore, it is being used to develop functional optical applications [12,15,16]. Furthermore, it has potential for use in various applications owing to its favorable appearance, which is similar to that of polymethylmethacrylate. This is attributed to its low refractive index, which is significantly different from that of bisphenol-A polycarbonate [12,15,17].

ISB-PC is highly polar, i.e., has a high solubility parameter. Therefore, to the best of our knowledge, there have been almost no reports on miscible/compatible polymer blends with ISB-PC, except in the case of reactive blending [18,19,20]. Triethyl citrate [21] and 4,4′-thiobis(6-*tert*-butyl-*m*-methyl phenol) [22], which are both low-molecular-weight compounds, are miscible with ISB-PC. Interestingly, they both act as antiplasticizers for ISB-PC, therefore increasing its modulus in the glassy state.

Herein, we focused on another polar polymer, i.e., poly(vinyl alcohol), which is not usually employed for thermo-processing. Because its melting point is higher than the degradation temperature of ISB-PC, severe thermal degradation occurs during processing in the molten state [23,24]. Therefore, specific techniques to reduce the crystallinity are required for thermo-processing [25,26,27,28,29]. One such technique, which has already been commercialized, involves the introduction of a small amount of butenediol as a comonomer. The resulting copolymer has a low melting point and can therefore be used for thermo-processing [30,31,32,33]. In the present study, we demonstrated that the addition of a small amount of this copolymer provides good adhesion with a commercially available poly(vinyl alcohol) glue, without the need for any surface treatment.

## 2. Results and Discussion

**Figure 1:**
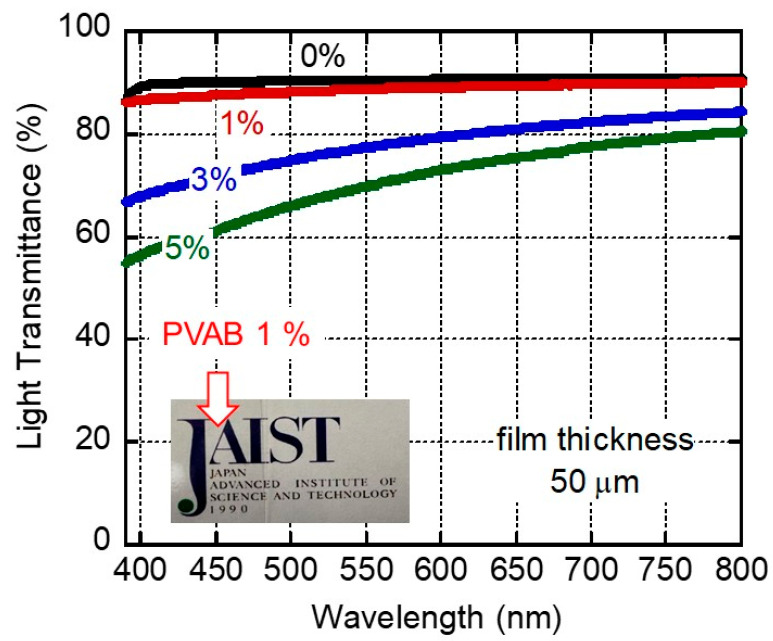
Light transmittance values of 50-μm-thick films of ISB-PC containing various amounts of PVAB. The figure also includes a photograph of a 50-μm-thick 1% PVAB film.

Figure 1 shows the light transmittance of compression-molded films with 0, 1, 3, and 5 wt.% of butenediol/vinyl alcohol copolymer (PVAB) as a function of wavelength. The film of pure ISB-PC, denoted as 0%, had high light transmittance values (>90%) in the visible wavelength. This high transmittance was attributable to the film’s low degree of surface reflection due to its low refractive index [15]. It is well known that pure polystyrene and bisphenol-A polycarbonate have light transmittance values of ca. 88% [34]. The 1% PVAB film was also highly transparent, as demonstrated by the photograph of a 50-μm-thick film shown in Figure 1. In fact, the transmittance values of the 1% PVAB film were almost similar to those of the pure ISB-PC, denoted as 0%. However, a slight decrease in the light transmittance suggested weak light scattering even in the 1% PVAB film, which will be discussed later.

Beyond 3%, the light transmittance values of the films clearly decreased, owing to light scattering. This must have been caused by the phase-separated structures of the films.

**Figure 2:**
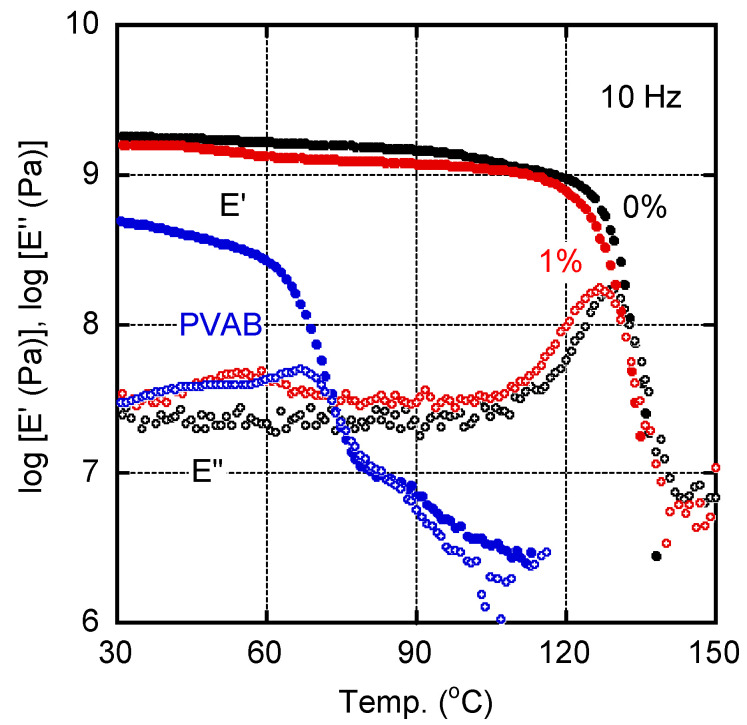
Temperature dependence of (closed symbols) tensile storage modulus *E*′ and (open symbols) loss modulus *E*″ at 10 Hz for the films of (black symbols) pure ISB-PC, i.e., 0%, and (red symbols) 1% PVAB. The data for the PVAB film were also plotted (blue symbols).

Figure 2 shows the temperature dependence of the tensile storage moduli *E*′ and loss moduli *E*″ for the sample films of pure ISB-PC (0%) and 1% PVAB. A pure PVAB film, prepared by compression molding, was also evaluated as a reference. The *E*′ values of PVAB in the glassy state were lower than those of the other samples, and decreased at approximately 60 °C owing to the glass-to-rubber transition. In the case of ISB-PC, we detected the typical viscoelastic behavior of a glassy polymer. The peak temperature in the *E*″ curve of ISB-PC, which was regarded as the glass transition temperature *T_g_*, was located at 128.8 °C. For the film with 1% PVAB, the peak temperature was shifted to 126.0 °C, suggesting mutual dissolution. Furthermore, the 1% PVAB film showed a weak but clear relaxation peak at around 55 °C, although the PVAB amount was only 1%. Considering the location of the peak temperature, this must be ascribed to the *T_g_* of PVAB, which will be discussed later with the SEM results.

**Figure 3:**
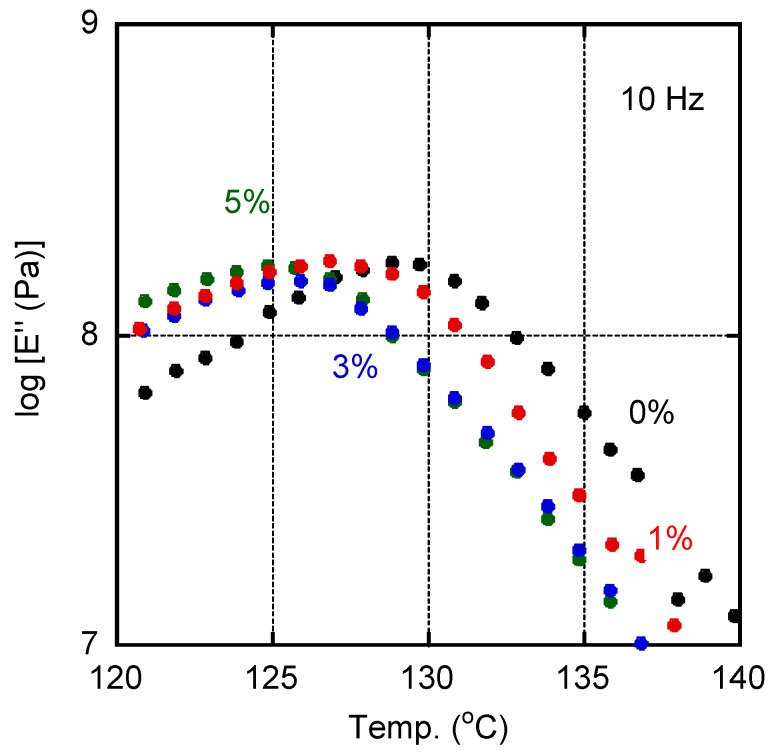
Temperature dependence of tensile loss modulus *E*″ at 10 Hz for the films containing various amounts of PVAB: (black symbols) 0%, (red symbols) 1%, (blue symbols) 3%, and (green symbols) 5%.

The *E*″ curves ascribed to the glass-to-rubber transition for films with various amounts of PVAB were shown in Figure 3. The addition of PVAB to the films was accompanied by a shift in the *T_g_*, indicating that a small amount of PVAB had dissolved in the ISB-PC. However, the *T_g_* shift was not so obvious when the PVAB content was beyond 1%.

**Figure 4:**
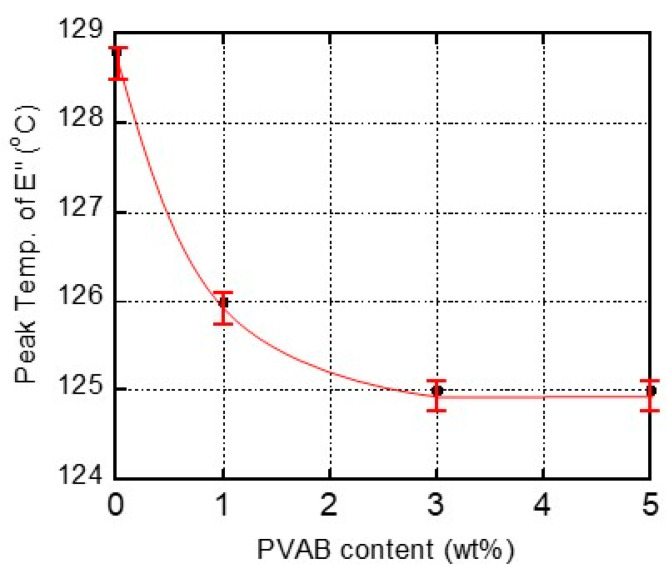
Relationship between the PVAB content and the peak temperature in the *E*″ curve ascribed to the glass-to-rubber transition of ISB-PC.

Figure 4 shows the relationship between the PVAB content and the peak temperature in the *E*″ curve ascribed to the *T_g_*. As seen in the figure, the films containing 3% and 5% PVAB had the same *T_g_* values, indicating that the maximum amount of PVAB dissolution in the ISB-PC must have been lower than 3%. This result corresponded with the transparency of the ISB-PC films.

**Figure 5:**
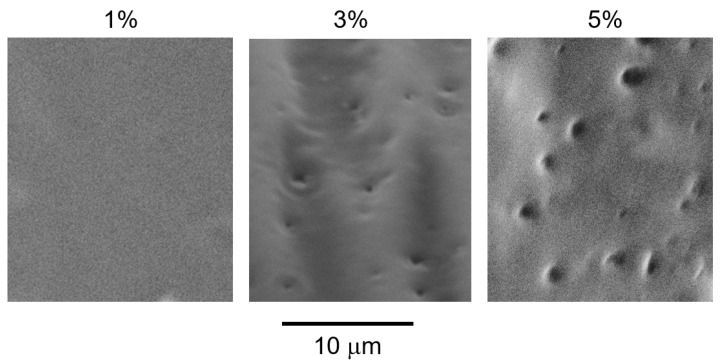
SEM images of the cut surfaces of the blended films with various PVAB contents, such as 1%, 3%, and 5%, after immersion in boiling water to remove PVAB.

Scanning electron microscopy (SEM) images of the cut surfaces of the blended films with various PVAB contents are shown in Figure 5. The samples were immersed in boiling water, and the voids represent the PVAB dispersion. The phase-separated structure was clearly visible in the blends containing 3% and 5% PVAB. However, such voids were not detected in the blend containing 1% PVAB.

Considering the results in Figure 1, Figure 2, Figure 3, Figure 4 and Figure 5, most PVAB chains (but not all of them) dissolved in ISB-PC when the PVAB content was 1%. Since phase separation, if any, was not obvious, the blend film with 1% PVAB was fairly transparent with no/less light scattering. Subsequently, we used the sample containing 1% PVAB, because the blends containing more than 3% lost their transparency.

The water droplet contact angles of the samples are summarized in Table 1. It is obvious that the contact angle, i.e., the surface tension, decreased markedly, even with only 1% PVAB. Such a large change would not be expected if the PVAB droplets existed as a dispersed phase. It is well known that various factors, such as surface roughness, affect the contact angle [35]. However, considering the experimental condition and the sample preparation method, the dissolution of a water-soluble material is responsible for this change. In other words, the PVAB chains on the film surface provide a hydrophilic nature to some degree.

**Figure 6:**
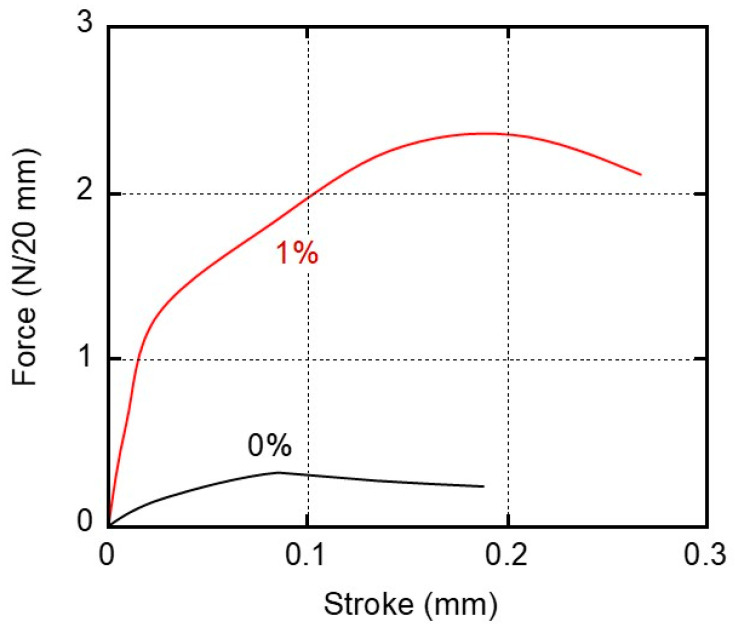
Stroke–force curves derived from the adhesive strength experiments using the sample films of (black line) 0% and (red line) 1% PVAB. The films employed were stored in a temperature/humidity chamber at 25 °C and 50% RH for 3 days before adhesion.

**Figure 7:**
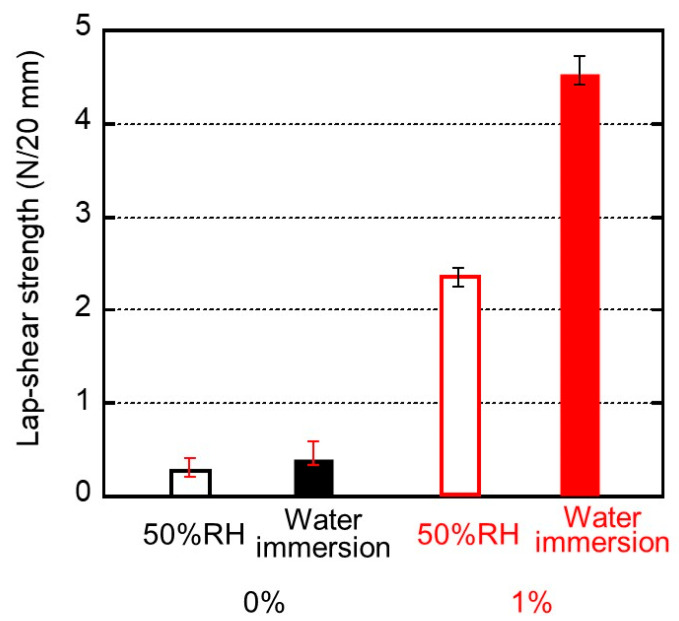
Lap-shear strengths and their standard deviation for the sample films of (black bars) 0% and (red bars) 1% PVAB. Before bonding, the films employed were (open bars) stored in a temperature/humidity chamber at 25 °C and 50% RH for 3 days and (filled bars) immersed in water at 25 °C for 3 days.

For the practical adhesion experiments, the sample specimens were prepared from films that had been kept at 25 °C and 50% relative humidity (RH) for 3 days. We kept other samples in distilled water (water immersion) at 25 °C for 3 days and used them for the experiments after wiping their surfaces. After laminating the films with the glue, the specimens were kept in a temperature–humidity chamber at 25 °C and 50% RH for 3 days prior to the lap-shear tests. Figure 6 shows the stroke–force curves of the samples kept at 25 °C and 50% RH for 3 days before laminating with the glue. The film width was 20 mm. It was found from the figure that the maximum forces were 0.3 N/20 mm for 0% and 2.3 N/20 mm for 1%, respectively (the data are summarized in Figure 7).

We evaluated the maximum force as lap-shear strength. The standard deviation was also inserted in the figure. Even the pure ISB-PC demonstrated some strength. As shown in Figure 7, the lap-shear strength of the blend film containing 1% PVAB was high and increased markedly after immersion in water. This experimental result indicates that the water content of the sample makes an important contribution to adhesion, although the strength was not improved enough by immersion in only water without PVAB. This will be discussed later. At least, this is the first report on good adhesion between a water-soluble glue and a conventional plastic without surface modification to the best of our knowledge.

**Figure 8:**
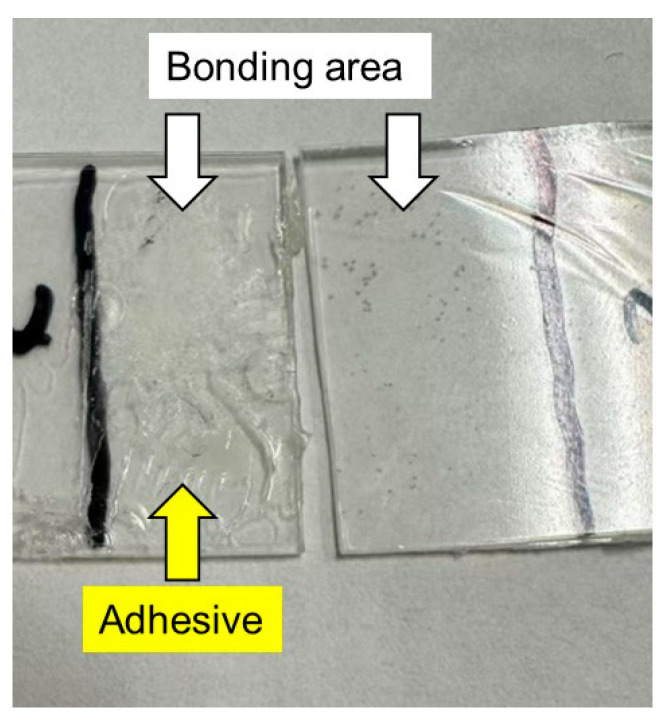
Photograph of sample films after the lap-shear experiment using films with 1% PVAB after being immersed in water at 25 °C for 3 days.

After the lap-shear tests, we examined the bonding area of each sample film and found the glue on one side of each specimen, as shown in Figure 8. In the figure, the film containing 1% PVAB was used after immersion in water. Therefore, it showed the highest lap-shear strength. The result demonstrated that adhesive bond failure occurred in all the samples.

There are several well-known models for adhesion of polymeric materials, such as (1) mechanical interlocking (or anchor effect), (2) interdiffusion (or entanglement couplings between layers), (3) molecular bonding such as covalent and ionic bonds, and (4) thermodynamic mechanisms at which interfacial tension plays an important role [1,2,36,37]. Among them, thermodynamic mechanisms, including dipole–dipole interaction, van der Waals force, and hydrogen bonds, must be important in the present study, especially for the sample with 1% PVAB after water immersion. In this study, adhesive bond failure was detected. This is because the adhesion occurs by secondary bonding, which is not strong enough to result in cohesive failure in the adhesive layer.

The enhancement of adhesion must be attributed to the dissolution of PVAB in the ISB-PC film. Water absorption, which may be enhanced by the PVAB addition, also contributed to the modification of surface properties, leading to good adhesion with the glue through hydrogen bonds and/or dipole–dipole interaction. Considering the experimental results, this technique will be applicable to improve the adhesion of ISB-PC with various types of water-soluble glues such as starch, dextrin, and gelatin. We are currently evaluating the adhesion properties using such glues to contribute to a sustainable society.

## 3. Materials and Methods

### 3.1. Sample Preparation

We used a randomly polymerized isosorbide-*co*-1,4-cyclohexanedimethanol copolycarbonate containing 70 wt.% isosorbide (denoted as ISB-PC) in the present study. Its melt flow rate was 10 g 10 min^−1^ at 230 °C under 2.16 kilogram-force (kgf), its density was 1360 kg m^−3^ at 23 °C, and its number- and weight-average molecular weights were 1.30 × 10^4^ Da and 3.79 × 10^4^ Da, respectively [31]. The basic properties of this copolycarbonate, including its entanglement molecular weight (3000) and melt density (1283.4 kg m^−3^ at 190 °C), have been reported in the literature [38]. We also used a copolymer of vinyl alcohol with a small amount of butenediol (AZF8035Q; Mitsubishi Chemical, Tokyo, Japan) [39], which was denoted as PVAB in the present study. Its melting point was 172 °C, its degree of polymerization was 300, and its degree of saponification was 98 mol.%. The details have been reported in the literature [32].

A conventional glue comprising aqueous poly(vinyl alcohol) (NA-150; YAMATO, Tokyo, Japan) was used for the adhesive experiments.

Various amounts of PVAB (0–5 wt.%) were added to the ISB-PC using a 30 cc internal batch mixer (Labo-Plastomill™; Toyo Seiki Seisaku-sho, Tokyo, Japan) at 180 °C for 5 min. The blade rotation speed was 30 rpm. Both the ISB-PC and the PVAB were dried under vacuum at 80 °C before melt-blending.

Flat films were prepared using a compression-molding machine at 180 °C under 10 MPa, and were subsequently quenched at 25 °C. The films were kept in a temperature/humidity-controlled chamber at 25 °C and 50% RH prior to the measurements.

### 3.2. Measurements

The light transmittance of each 50-μm-thick film was measured using a UV–vis spectrometer at 25 °C (Lambda 25; PerkinElmer, Waltham, MA, USA). The wavelength range was 380–800 nm.

The temperature dependence of the dynamic tensile modulus was determined using a dynamic mechanical analyzer (Rheogel-4000; UBM, Muko, Japan). Rectangular specimens (0.5 mm thick) were cut from the compression-molded films. Each was 5 mm wide and had a gauge length of 20 mm. The measurements were obtained at a constant heating rate of 2 °C min^−1^. The frequency applied was 10 Hz to obtain reliable data from this machine.

The contact angle regarding a drop of distilled water (1.0 μL) was measured using a contact-angle meter (Drop Master DM-501; Kyowa Interface Science, Saitama, Japan) at 23 °C. Since the droplet size (ca. 1000 μm) was much larger than the phase-separated structure (ca. 1 μm in the 3% PVAB film), phase separation, if any, did not affect the result. The measurements were obtained five times, and the average values were calculated.

The cut surface of each compression-molded blend film was examined by SEM (TM3030Plus; Hitachi, Tokyo, Japan) to determine its morphology. Before examination, each specimen with a cut surface was immersed in boiling water for 30 min to remove the PVAB component. After drying, the samples were sputtered with Pt/Pd.

**Figure 9:**
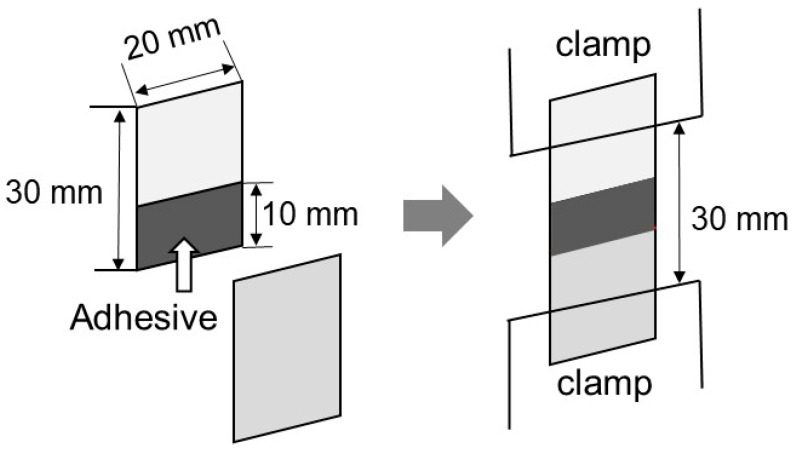
Schematic illustration of the lap-shear tests.

The adhesion property was evaluated at 25 °C by lap-shear tests. The sample films (20 mm wide, 30 mm long, and 0.5 mm thick) were cut from the compression-molded films. Some of the samples were kept in a temperature/humidity chamber at 25 °C and 50% RH for 3 days, and the others were immersed in water at 25 °C for 3 days. Two films that had been stored under the same conditions were attached together, as shown in Figure 9. The glue was applied between the films over an area of 10 mm × 20 mm to a thickness of approximately 100 μm. After bonding, the sample specimens were kept in the temperature/humidity-controlled chamber at 25 °C and 50% RH for 3 days before the measurements were obtained. The lap-shear strength was evaluated using a universal testing machine (EX-LX HS; Shimadzu, Kyoto, Japan). The initial distance between the clamps was 30 mm, and one of them moved at a constant speed of 1 mm min^−1^.

## 4. Conclusions

Although there have been only limited reports on miscible polymer blends comprising ISB-PC, we found that a small amount of PVAB was miscible with ISB-PC. The results were supported by light transmittance, i.e., transparency, dynamic mechanical properties in the solid state, and SEM observation. Furthermore, the contact angle regarding a water droplet decreased markedly following the addition of 1% PVAB. This was owing to the dissolution of the PVAB chains in the ISB-PC, although the maximum content of PVAB for dissolution in the ISB-PC was less than 1%. It should be noted that the blend of ISB-PC with 1% PVAB had good adhesion regarding an aqueous glue comprising poly(vinyl alcohol). Considering that the glue is biodegradable, it will be useful for various applications.

## Figures and Tables

**Table 1:** Water droplet contact angles.

	0%	1%
Contact angle	74.6	68.8
(degree)	(72.4–78.1)	(66.7–71.3)

The numbers in parentheses represent the experimental error.

## Data Availability

Data will be available upon request.

## References

[1] Ebnesajjad S., Landrock A.H. (2015). Adhesins Technology Handbook.

[2] Adams R.D. (2021). Adhesive Bonding: Science, Technology and Applications.

[3] Mitrano D.M., Wohlleben W. (2020). Microplastic regulation should be more precise to incentivize both innovation and environmental safety. Nat. Commun..

[4] Lambert S., Wagner M. (2017). Environmental performance of bio-based and biodegradable plastics: The road ahead. Chem. Soc. Rev..

[5] Ghaffarianhoseini A., AlWaer H., Omrany H., Ghaffarianhoseini A., Alalouch C., Clements-Croome D., Tookey J. (2018). Sick building syndrome: Are we doing enough?. Archit. Sci. Rev..

[6] Kawai F., Hu X. (2009). Biochemistry of microbial polyvinyl alcohol degradation. Appl. Microbiol. Biotechnol..

[7] Huang D., Hu Z., Ding Y., Zhen Z., Lu B., Ji J., Wang G. (2019). Seawater degradable PVA/PCL blends with water-soluble polyvinyl alcohol as degradation accelerator. Polym. Degrad. Stab..

[8] Alonso-Lopez O., Lopez-Ibanez S., Beiras R. (2021). Assessment of toxicity and biodegradability of poly(vinyl alcohol)-based materials in marine water. Polymers.

[9] Vineeth S.K., Gadhave R.V. (2024). Corn starch blended polyvinyl alcohol adhesive chemically modified by crosslinking and its applicability as polyvinyl acetate wood adhesive. Polym. Bull..

[10] Kristufek T.S., Kristufek S.L., Link L.A., Weems A.C., Khan S., Lim S.M., Wooley K.L. (2016). Rapidly-cured isosorbide-based cross-linked polycarbonate elastomers. Polym. Chem..

[11] Wu F., Pu Z., Hou H., Li X., Zhu R., Wang X., Zhong J. (2023). Comparison of the properties of bioderived polycarbonate and traditional bisphenol-A polycarbonate. J. Polym. Res..

[12] Chu J., Wang H., Zhang Y., Li Z., Zhang Z., He H., Xu F. (2022). Design and synthesis of gradient-refractive index isosorbide-based polycarbonates for optical uses. React. Funct. Polym..

[13] Li C., Long X., Wang Q., Li J., Zhang H., Wang G. (2022). Studies on synthesis and optical properties of poly(isosorbide-co-1,4-cyclohexanedimethanol) carbonate. J. Polym. Res..

[14] Aricò F., Tundo P. (2016). Isosorbide and dimethyl carbonate: A green match. Beilstein J. Org. Chem..

[15] Miyashita M., Yamaguchi M. (2020). Effect of water absorption on the structure and properties of isosorbide-based polycarbonate. Polymer.

[16] Li Z., Wang H., Yan H., Wang H., Zhang Z., Zhang Y., Xu F. (2024). Design and synthesis of optical biobased polycarbonates with high refractive index and low birefringence. Ind. Eng. Chem. Res..

[17] Sawada R., Ando S. (2022). Colorless, low dielectric, and optically active semialicyclic polyimides incorporating a biobased isosorbide moiety in the main chain. Macromolecules.

[18] Park S.A., Choi J., Ju S., Jegal J., Lee K.M., Hwang S.Y., Park J. (2017). Copolycarbonates of bio-based rigid isosorbide and flexible 1,4-cyclohexanedimethanol: Merits over bisphenol-A based polycarbonates. Polymer.

[19] Lai W., Wu G. (2019). Reactive blending and transesterification-induced degradation of isosorbide-based polycarbonate blends. Polym. Degrad. Stab..

[20] Park S.A., Eom Y., Jeon H., Koo J.M., Lee E.S., Jegal J., Park J. (2019). Preparation of synergistically reinforced transparent bio-polycarbonate nanocomposites with highly dispersed cellulose nanocrystals. Green Chem..

[21] Han R., Kida T., Yamaguchi M. (2024). Antiplasticizing effect of triethyl citrate on an isosorbide-based polycarbonate. J. Polym. Res..

[22] Su L., Lai W., Yan J., Wu G. (2017). Small-molecule-induced miscibility of isosorbide-based polycarbonate with bisphenol A polycarbonate. J. Appl. Polym. Sci..

[23] Holland B.J., Hay J.N. (2001). The thermal degradation of poly(vinyl alcohol). Polymer.

[24] Alexy P., Lacik I., Simkova B., Bakos D., Pronayava N., Liptaj T., Hanzelova S., Varosova M. (2004). Effect of melt processing on thermo-mechanical degradation of poly(vinyl alcohol)s. Polym. Degrad. Stab..

[25] Nishio T., Kani S., Gotoh K., Nakamae K. (2002). Melt processing of poly(vinyl alcohol) through blending with sugar pendant polymer. Polymer.

[26] Chen N., Li L., Wang Q. (2007). New technology for thermal processing of poly(vinyl alcohol). Plast. Rubber Compos..

[27] Tian H., Yao Y., Ma S., Zhang X., Xiang A. (2017). Effect of sorbitol plasticizer on the structure and properties of melt processed polyvinyl alcohol films. Food Sci..

[28] Nishikawa R., Aridome N., Ojima N., Yamaguchi M. (2020). Structure and properties of fiber-reinforced polypropylene prepared by direct incorporation of aqueous solution of poly(vinyl alcohol). Polymer.

[29] Saari R.A., Nasri M.S., Kida T., Yamaguchi M. (2021). Impact of magnesium salt on the mechanical and thermal properties of poly(vinyl alcohol). Polymers.

[30] Jung B.N., Kang D.H., Shim J.K., Hwang S.W. (2019). Physical and mechanical properties of plasticized butenediol vinyl alcohol copolymer/thermoplastic starch blend. J. Vinyl Addit. Technol..

[31] Xing J., Wang R., Sun S., Shen Y., Liang B., Xu Z. (2023). Morphology and properties of polylactic acid composites with butenediol vinyl alcohol copolymer formed by melt blending. Molecules.

[32] Yamaguchi M., Shu W., Kimura T., Vo H.G.D., Kida T., Mori T., Miyamoto A. (2023). Anomalous postprocessing dimensional change of injection-molded products composed of poly(lactic acid) and poly(vinyl alcohol). ACS Appl. Polym. Mater..

[33] Yu D., Yang Q., Zhou X., Guo H., Li D., Li H., Deng B., Liu Q. (2023). Structure and properties of polylactic acid/butenediol vinyl alcohol copolymer blend fibers. Intern. J. Biolog. Macoromol..

[34] Imai Y., Terahara A., Hakuta Y., Matsui K., Hayashi H., Ueno N. (2009). Transparent poly(bisphenol A carbonate)-based nanocomposites with high refractive index nanoparticles. Eur. Polym. J..

[35] Good R.J. (1992). Contact angle, wetting, and adhesion: A critical review. J. Adh. Sci. Technol..

[36] Awaja F., Cilbert M., Kelly G., Fox B., Pigram P.J. (2009). Adhesion of polymers. Progr. Polym. Sci..

[37] Baldan A. (2012). Adhesion phenomena in bonded joints. Int. J. Adhes. Adhes..

[38] Han R., Kida T., Yamaguchi M. (2023). Viscoelastic properties of copolycarbonates comprising isosorbide and 1, 4-cyclohexanedimethanol. Colloid Polym. Sci..

[39] Tomita M., Yamauchi Y., Kuroki H. (2009). Method for Producing Polyvinyl Alcohol Resin Having 1,2-diol Structure in Side Chain.

